# Atherogenic Lipoprotein Burden, Metabolic Stress and Immune Activation Associated with Coronary Atherosclerosis in Patients with Psoriasis

**DOI:** 10.3390/ijms27052353

**Published:** 2026-03-03

**Authors:** Lazar Djukanovic, Dusan Skiljevic, Milos Nikolic, Marija Malinic, Svetlana Popadic, Oliver Radmili, Vladimir Cvetic, Nina Rajovic, Natasa Milic, Lidija Savic, Lidija Maslac, Milika Asanin, Sanja Stankovic, Ratko Lasica

**Affiliations:** 1 Department of Cardiology, University Clinical Center of Serbia, 11000 Belgrade, Serbia; lazardjukanovic08@gmail.com (L.D.); lidijamaslac98@gmail.com (L.M.); 2 Faculty of Medicine, University of Belgrade, 11000 Belgrade, Serbia; dusanskiljevic@yahoo.com (D.S.); milos.nikolic@med.bg.ac.rs (M.N.); prof.svetlana.popadic@gmail.com (S.P.); drvladimircvetic@gmail.com (V.C.); nina94rajovic@gmail.com (N.R.); silly_stat@yahoo.com (N.M.); lidijasavic2007@gmail.com (L.S.); masanin2013@gmail.com (M.A.); 3 Clinic of Dermatology and Venereology, University Clinical Center of Serbia, 11000 Belgrade, Serbia; marija.malinic@gmail.com; 4 Clinic for Vascular and Endovascular Surgery, University Clinical Center of Serbia, 11000 Belgrade, Serbia; oliver_radmili@yahoo.com; 5 Institute of Medical Statistics and Informatics, Faculty of Medicine, University of Belgrade, 11000 Belgrade, Serbia; 6 Department of Cardiology, Emergency Center, University Clinical Center of Serbia, 11000 Belgrade, Serbia; 7 Center for Medical Biochemistry, University Clinical Center of Serbia, 11000 Belgrade, Serbia; 8 Faculty of Medical Sciences, University of Kragujevac, 34000 Kragujevac, Serbia

**Keywords:** psoriasis, atherosclerosis, remnant cholesterol, immunoglobulin A, metabolic syndrome

## Abstract

Psoriasis is a chronic inflammatory disease associated with an increased cardiovascular risk (CVR). The mechanisms linking psoriasis to coronary atherosclerosis have not yet been fully elucidated. A dynamic interplay between metabolic disturbances, immune mechanisms, and elevated atherogenic lipoprotein particles may contribute to the accelerated development of atherosclerosis. Patients with psoriasis (*n* = 104) without known coronary artery disease underwent coronary computed tomography angiography (CCTA) to detect subclinical coronary atherosclerosis. Clinical data, metabolic parameters and indices, lipid fractions including remnant cholesterol, and immunological markers (immunoglobulin A- IgA) were analyzed. Associations with CT-confirmed coronary stenosis were assessed using univariate and multivariate logistic regression models. Patients with coronary atherosclerosis exhibited a more adverse metabolic and lipid profile. Remnant cholesterol emerged as a strong independent predictor of coronary stenosis. Elevated IgA levels were associated with the presence of coronary atherosclerosis, suggesting a potential role of immune activation that extends beyond general systemic inflammation. Longer duration of psoriasis correlated with the presence of coronary atherosclerosis, highlighting the importance of cumulative inflammatory burden. Our findings indicate that subclinical coronary atherosclerosis in patients with psoriasis is closely associated with an immuno-metabolic risk profile encompassing atherogenic lipoprotein fractions and immune activation. These results underscore the need for a broader approach to cardiovascular risk assessment in this population, extending beyond the evaluation of traditional cardiovascular risk factors alone.

## 1. Introduction

Psoriasis is a common chronic inflammatory disease with an estimated prevalence ranging from 0.9% to 8.5% in adults and approximately 2.1% in children [1]. It most commonly manifests as well-demarcated erythematous plaques covered with silvery scales, predominantly affecting extensor surfaces of the extremities, the scalp, and the lumbosacral region. Plaque psoriasis accounts for approximately 85–90% of all psoriasis cases [2,3]. Although cutaneous manifestations represent the most prominent clinical feature, contemporary understanding of psoriasis extends beyond the concept of an isolated dermatological disorder, defining it as a chronic, immune-mediated inflammatory condition with systemic consequences. Numerous studies have demonstrated an association between psoriasis and a wide spectrum of comorbidities, most notably cardiovascular and metabolic disorders, psoriatic arthritis, psychiatric conditions, and hepatometabolic disease [4,5,6,7].

Atherosclerosis is a complex, multifactorial process driven by the interplay of chronic inflammation, immune cell activation, endothelial dysfunction, and lipid accumulation [8]. Although low-density lipoprotein cholesterol (LDL-C) has traditionally been regarded as the primary lipid driver of atherosclerosis, accumulating evidence highlights the importance of a broader spectrum of atherogenic lipoproteins, including remnant cholesterol. Remnant lipoproteins comprise triglyceride-rich, apolipoprotein B-containing lipoproteins, including very-low-density lipoproteins (VLDL), intermediate-density lipoproteins (IDL), and chylomicron remnants [9]. Unlike LDL particles, remnant lipoproteins can directly penetrate the subendothelial space without prior oxidative modification, where they become retained within the arterial intima and promote inflammatory responses. Remnant-C has been associated with endothelial dysfunction, monocyte and macrophage activation, and increased production of proinflammatory cytokines, thereby directly contributing to the formation and progression of atherosclerotic plaques [10]. Epidemiological and genetic studies have demonstrated that elevated remnant cholesterol concentrations are strongly associated with the risk of atherosclerotic cardiovascular disease, independent of LDL cholesterol, indicating that remnant lipoproteins represent a clinically relevant and often underappreciated lipid pathway in atherogenesis [11,12].

Immunological mechanisms, encompassing both innate and adaptive immune responses, play a central role in the initiation and progression of atherosclerotic plaques and are particularly relevant in the context of systemic inflammatory diseases such as psoriasis [13]. Innate immunity involves endothelial cells, monocytes, and macrophages, which respond to modified lipoproteins and inflammatory signals by initiating local inflammatory responses, leukocyte recruitment, and foam cell formation. Adaptive immunity includes activation of T lymphocytes, particularly Th1 and Th17 subsets, whose secretion of proinflammatory cytokines further amplifies inflammation, affects vascular smooth muscle cell function, and contributes to plaque destabilization [13]. A similar pattern of adaptive immune activation, characterized by dominant Th1 and Th17 involvement, is also observed in psoriatic skin lesions. In addition to T-cell-mediated cellular immunity, atherosclerosis is accompanied by activation of humoral immune responses, reflected by increased production of circulating immunoglobulins. In systemic inflammatory diseases such as psoriasis, chronic immune activation is not confined to the skin but exerts systemic effects, creating a proatherogenic milieu that accelerates vascular injury and atherosclerotic progression. Levels of immunological biomarkers, including circulating immunoglobulins, cytokines, and other inflammatory mediators, may therefore reflect both disease activity and vascular risk. Nevertheless, their role as predictors of atherosclerosis in patients with psoriasis remains insufficiently clarified. Previous studies investigating the association between immunoglobulin A (IgA) and atherosclerosis have demonstrated a significant relationship with cardiovascular risk and atherosclerotic outcomes [14]. However, the potential predictive role of IgA in atherosclerosis among patients with psoriasis has not yet been specifically investigated.

Patients with psoriasis exhibit a higher prevalence of conventional risk factors for atherosclerotic disease, including diabetes mellitus, arterial hypertension, hyperlipidemia, smoking, and increased body mass index (BMI), compared with the general population [15]. Both metabolic syndrome and its individual components are more frequently observed in patients with psoriasis. Previous studies have shown that the prevalence of metabolic syndrome is substantially higher in patients with psoriasis than in healthy controls (39.3% vs. 17.1%; OR = 3.13) [16]. Metabolic indices derived from routine biochemical parameters, particularly those reflecting triglyceride levels and insulin resistance, have emerged as readily accessible tools for cardiovascular risk stratification. Among these, the triglyceride–glucose (TyG) index has gained particular attention as a reliable surrogate marker of insulin resistance that integrates disturbances in glucose and lipid metabolism. However, despite growing evidence, the predictive value of metabolic indices for subclinical atherosclerosis in patients with psoriasis, especially in the context of their interaction with immunological and lipid-related mechanisms, remains insufficiently explored.

The association between psoriasis and CVD spans the entire cardiovascular continuum, ranging from microvascular dysfunction and asymptomatic atherosclerosis to subclinical coronary artery disease and severe clinical outcomes, including acute myocardial infarction, stroke, heart failure, and increased cardiovascular mortality [17,18,19]. Although traditional cardiovascular risk factors are more prevalent in patients with psoriasis, an increasing body of literature indicates that systemic inflammation and immune system activation represent additional, clinically relevant contributors to atherogenesis in this population, leading to measurable increases in vascular inflammation and subclinical coronary disease [20]. Accordingly, the 2021 European Society of Cardiology (ESC) guidelines and the 2019 ACC/AHA guidelines for cardiovascular disease prevention have recognized psoriasis as a risk-modifying CVR, alongside conventional risk factors, in the assessment of adverse cardiovascular events [21,22].

Despite contemporary therapeutic strategies targeting traditional cardiovascular risk factors, such as hypertension, obesity, dyslipidemia, and smoking cessation, atherosclerotic disease continues to progress in a substantial proportion of patients. This discrepancy suggests that conventional risk factors do not fully capture the complex pathophysiology of atherogenesis, particularly in patients with psoriasis. Consequently, increasing attention has been directed toward alternative and complementary mechanisms, including cumulative inflammatory burden, subclinical immune processes, atherogenic lipoprotein fractions not routinely assessed in standard lipid profiles, and pronounced metabolic dysregulation, all of which may collectively contribute to the development and progression of atherosclerotic disease.

Identification of circulating biochemical predictors of atherosclerosis could improve early cardiovascular risk assessment and provide deeper insight into psoriasis-specific mechanisms of atherogenesis. Therefore, the aim of this study was to investigate the association between atherogenic lipid parameters, immunological markers, and metabolic biomarkers with the presence of atherosclerosis in patients with psoriasis, with particular emphasis on identifying independent biochemical predictors.

## 2. Results

A total of 104 patients diagnosed with psoriasis were included in the study. The majority of participants were male (71.2%), with a mean age of 47.9 ± 14.1 years. More than half of the patients were married (55.4%) and had completed secondary education (67.3%).

Among the total study population, 93 patients underwent CT scanning for stenosis assessment. Four patients withdrew from the study prior to the scheduled CCTA examination; three patients were subsequently diagnosed with another inflammatory disease; two patients developed an additional dermatological condition; one female patient was diagnosed with pregnancy; and one patient experienced a significant deterioration of renal function that was not present at the initial evaluation. Baseline clinical and laboratory characteristics did not differ significantly between patients included in the final analysis (CCTA performed) and those excluded before the scheduled CCTA examination. This indicates comparability of the two groups of patients. Patients with CT-confirmed stenosis were significantly older (*p* < 0.001), more frequently male (*p* = 0.005), had a higher waist circumference (*p* = 0.012), were more often ex-smokers (*p* = 0.031), had a longer smoking history (*p* = 0.005). Regarding comorbidities, patients with CT-confirmed stenosis more frequently had hypertension (*p* = 0.005), hyperlipidemia (*p* = 0.017), and hypertriglyceridemia (*p* < 0.001). In terms of treatment modalities, these patients more often received prior antihypertensive therapy (*p* < 0.001) and statins (*p* = 0.005). Sociodemographic characteristics, comorbidities, and treatment modalities according to atherosclerotic changes on CT in patients with psoriasis are presented in detail in Table 1.

Patients with CT-confirmed stenosis were significantly older at psoriasis onset (*p* = 0.001), had a longer disease duration (*p* = 0.008), lower Dermatology Life Quality Index (DLQI) scores (*p* = 0.005) and more frequently met the criteria for metabolic syndrome (*p* = 0.001). Characteristics of psoriasis according to CT-confirmed coronary stenosis are presented in Table 2.

**Figure 1:**
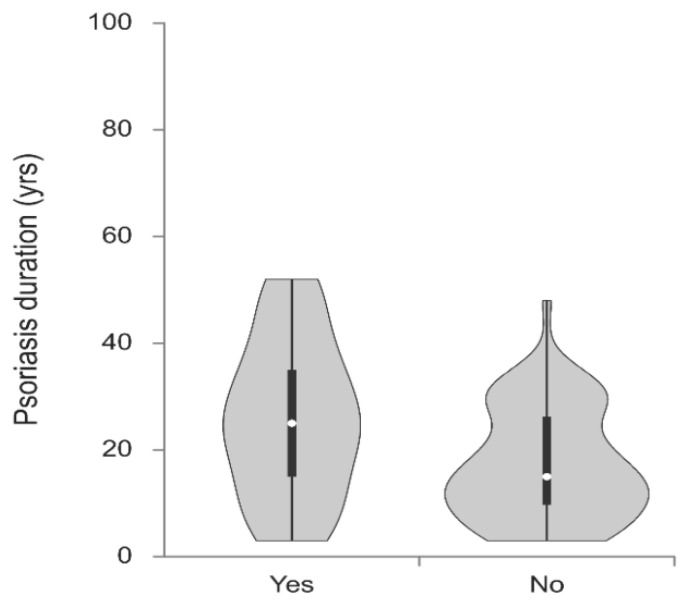
Violin plot showing psoriasis duration (yrs) according to CT-confirmed stenosis in patients with psoriasis.

The violin plot demonstrates that both the mean and median duration of psoriasis were longer in patients with CCTA-confirmed coronary stenosis compared with those without stenosis. At the same time, the distribution of psoriasis duration was wider in patients without stenosis, particularly in the range of shorter disease duration, indicating greater heterogeneity within this group (Figure 1).

Patients with CT-confirmed stenosis were more likely to receive systemic therapy (*p* = 0.015). No other treatment modalities differed significantly between the groups (*p* > 0.05). Differences in psoriasis therapy according to the presence of CT-confirmed stenosis are presented in Table 3.

Significant laboratory parameters according to the presence of CT-confirmed stenosis are presented in Table 4.

**Figure 2:**
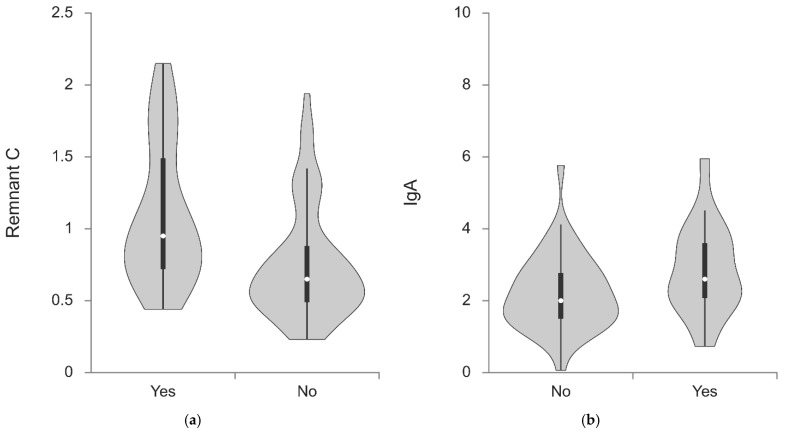
Violin plot showing Remnant C values (**a**) and IgA values (**b**) according to CT-confirmed stenosis in patients with psoriasis.

Violin plot (a) illustrates a rightward shift in the distribution of remnant cholesterol (Remnant C) toward higher values in patients with CT-confirmed coronary stenosis, accompanied by a wider range of values compared with the non-stenosis group. In contrast, patients without stenosis exhibit a narrower distribution with a predominant concentration of lower Remnant C values. Violin plot (b) demonstrates that IgA levels are shifted toward higher values in patients with CCTA-confirmed stenosis compared with those without stenosis, with a higher central tendency observed in the stenosis group. The IgA distribution in patients with stenosis spans a higher value range, whereas in patients without stenosis, IgA values are predominantly concentrated within the lower to mid-range (Figure 2).

Univariate logistic regression model with CT-confirmed stenosis in patients with psoriasis as dependent variable is presented in Table 5.

Taking into account metabolic parameters, immunologic and inflammatory markers, and lipid parameters that were significant in the univariate logistic regression analysis, two multivariate logistic regression models were constructed. The variable Remnant C was categorized into tertiles for inclusion in the models.

In Model 1, patients in the third tertile of Remnant C had significantly higher odds of CT-confirmed stenosis compared with those in the first tertile (OR = 14.095, 95% CI: 2.241–88.660, *p* = 0.005), whereas the second tertile of Remnant C showed no statistical significance (*p* = 0.181). In addition, higher IgA (OR =1.863, 95% CI: 1.043–3.325, *p* = 0.035) and longer psoriasis duration (OR = 1.081, 95% CI: 1.019–1.147, *p* = 0.010) were significant predictors of CT-confirmed stenosis in multivariate logistic regression analysis.

**Figure 3:**
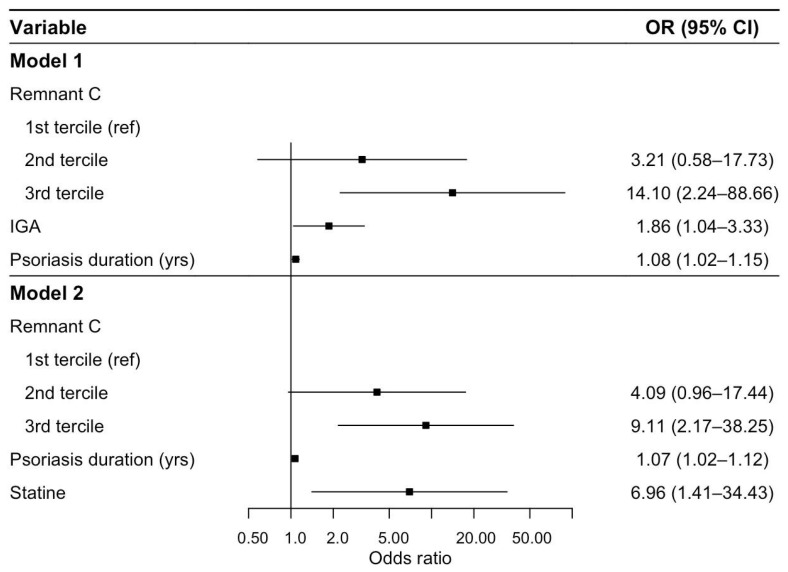
Forest plot of odds ratios from multivariable logistic regression models.

In Model 2, the third tertile of Remnant C had significantly higher odds of CT-confirmed stenosis compared with those in the first tertile (OR = 9.112, 95% CI: 2.171–38.249, *p* = 0.003), while the second tertile of Remnant C showed no statistical significance (*p* = 0.057). Longer psoriasis duration (OR = 1.069, 95% CI: 1.023–1.117, *p* = 0.003) and statin therapy were significantly associated with higher odds of CT-confirmed stenosis (OR = 6.964, 95% CI: 1.409–34.426, *p* = 0.017). Results of the multivariate logistic regression analysis with CT-confirmed stenosis as the dependent variable are presented in Figure 3.

## 3. Discussion

Several large population-based studies have demonstrated that the relative cardiovascular risk (CVR) associated with psoriasis is most pronounced in younger patients, in whom traditional cardiovascular risk factors are often less dominant [23,24]. In our study, older age represented a strong and statistically significant factor associated with the presence of atherosclerosis; however, it is important to emphasize that the studied cohort included a substantial proportion of younger patients. Among participants who underwent CCTA, nearly half (45.1%) were younger than 40 years, including 12.7% younger than 30 years. Despite this relatively young age profile, atherosclerosis was detected in 31.2% of the overall population, and 34.5% of patients with atherosclerosis fulfilled criteria for premature disease according to current definitions (<55 years in men and <65 years in women).

Our findings are consistent with the established role of age as a major contributor to CVR; however, patients with psoriasis may exhibit an increased burden of premature atherosclerosis, consistent with previous reports suggesting psoriasis as a potential independent proatherogenic condition. These observations are consistent with previous studies reporting an increased prevalence of premature atherosclerosis among patients with psoriasis [25,26].

CCTA-confirmed atherosclerosis in our study was significantly more frequent in men than in women. This finding aligns with well-established sex-related differences in the development and progression of atherosclerosis, with women generally developing atherosclerotic changes less frequently and later in life compared with men [17,25].

Several studies and meta-analyses have shown that patients with psoriasis are more frequently long-term smokers, and that smoking correlates with more pronounced systemic inflammation and more severe clinical disease [27,28]. In our cohort, 58.1% of patients were active smokers, and prior smoking status differed significantly between patients with and without CT-confirmed stenosis (*p* = 0.031). Importantly, cumulative smoking exposure showed a stronger association with atherosclerosis: patients with stenosis had a longer smoking history (25 [15, 16, 17, …, 40] vs. 15 [9, 10, 11, …, 20] years; *p* = 0.005), and smoking duration remained a significant related to the presence of atherosclerosis in univariable analysis (OR 1.058; 95% CI 1.014–1.104; *p* = 0.013). The association between smoking and atherosclerosis reflects oxidative stress, reactive oxygen species generation, and endothelial dysfunction; in the context of psoriasis, smoking further amplifies systemic inflammation, creating a synergistic proatherogenic effect alongside traditional cardiovascular risk factors [28,29].

### 3.1. Metabolic Parameters

In our study, metabolic parameters differed significantly according to the presence of coronary atherosclerosis, with a pronounced metabolic imbalance observed in patients with stenosis. Higher fasting glucose, HbA1c levels, and a higher TyG index in patients with confirmed atherosclerosis indicate more pronounced insulin resistance in this group. Even mildly elevated glucose and HbA1c levels in non-diabetic populations are associated with increased CVR and are pathophysiologically linked to oxidative stress, formation of advanced glycation end-products, and endothelial dysfunction, thereby accelerating plaque development and progression [30,31]. HbA1c values as low as 5.5–6.4% have been associated with adverse cardiovascular events even in individuals without diabetes [32].

The TyG index is a simple, routinely available surrogate marker of insulin resistance that correlates positively with the gold standard for insulin sensitivity assessment, the euglycemic–hyperinsulinemic clamp [33,34]. Lee et al. demonstrated that a higher TyG index is associated with an increased risk of coronary stenosis in asymptomatic patients with type 2 diabetes [35]. The association between the TyG index and coronary atherosclerosis has also been demonstrated in individuals without diabetes and in patients with chronic systemic inflammatory diseases such as rheumatoid arthritis [36,37,38]. A meta-analysis based on reanalysis of 41 studies showed that individuals with the highest TyG values had a significantly higher risk of developing coronary artery disease compared with those with the lowest values (OR ≈ 1.94; 95% CI 1.20–3.14; *p* = 0.007), and that a higher TyG index was associated with a markedly increased likelihood of stenotic coronary lesions (OR ≈ 3.49; 95% CI 1.71–7.12; *p* = 0.0006) [39]. Although the TyG index has been linked to atherosclerotic disease in patients with diabetes and certain inflammatory conditions, data on its role in psoriasis remain limited and largely confined to metabolic aspects of the disease. In our study, univariable analysis demonstrated that elevated TyG values were associated with 3.35-fold higher odds of coronary atherosclerosis compared with patients with normal TyG values. These findings suggest that the TyG index may serve as a useful marker of a metabolic–inflammatory phenotype associated with coronary atherosclerosis in patients with psoriasis.

Although BMI was higher in patients with atherosclerosis (31.4 ± 6.2 vs. 28.7 ± 6.7 kg/m^2^), it did not emerge as a statistically significant predictor, in contrast to waist circumference (107.8 ± 14.7 vs. 98.5 ± 16.8 cm; *p* = 0.012), highlighting the potential importance of central obesity in relation to coronary risk. Central adiposity more accurately reflects visceral fat burden and thus better captures atherogenic and prognostic risk in patients with psoriasis [40,41]. In our cohort, the prevalence of metabolic syndrome was 39.8%, and it was significantly more frequent among patients with atherosclerosis (65.5% vs. 28.1%; *p* = 0.001), who also exhibited a higher metabolic score (3 [2, 3, 4] vs. 2 [1, 2, 3]; *p* < 0.001). Previous studies have shown that metabolic syndrome is more prevalent in patients with more severe psoriasis, with adjusted ORs of ~1.22 for mild and ~1.98 for severe psoriasis compared with controls [42]. Meta-analyses by Mottillo et al. corroborate our findings, indicating that metabolic syndrome approximately doubles the risk of coronary artery disease [43]. The presence of metabolic syndrome is clearly associated with atherosclerotic disease both in patients with psoriasis and in the general population [44,45].

### 3.2. Atherogenic Potential

Lipid profiles differed significantly between patients with and without CT-confirmed atherosclerosis, indicating a pronounced atherogenic phenotype in the stenosis group. Prior hyperlipidemia, particularly hypertriglyceridemia, was more frequent among patients with atherosclerosis and was accompanied by more frequent statin use (24.1% vs. 4.7%; *p* = 0.005). Although total cholesterol, HDL-C, and LDL-C did not differ significantly, patients with stenosis exhibited higher triglyceride and ApoB levels with preserved ApoA1 values. Accordingly, the atherogenic coefficient, reflecting triglyceride-rich dyslipidemia, was significantly elevated (*p* = 0.036).

Remnant C was particularly prominent, with significantly higher levels in patients with coronary atherosclerosis (0.95 [0.72–1.49] vs. 0.65 [0.49–0.89] mmol/L; *p* < 0.001), highlighting the potential importance of remnant lipoproteins as an atherogenic pathway independent of LDL cholesterol. Univariable analysis demonstrated a strong association between elevated Remnant C and atherosclerosis (OR 5.502; 95% CI 1.910–15.848; *p* = 0.004). In multivariable logistic regression, the highest tertile of Remnant C remained independently associated with the presence of coronary atherosclerosis after adjustment for psoriasis duration, immunological parameters, and statin therapy. The persistence of this association across models suggests a potential independent contribution of remnant lipoproteins to atherosclerotic burden in this population.

Statin therapy was independently associated with CT-confirmed stenosis, most likely reflecting confounding by indication, whereby statins are preferentially prescribed to individuals with higher baseline risk or recognized dyslipidemia; thus, in a cross-sectional design, statin use may act as a marker of higher-risk phenotype rather than a harmful effect of therapy itself.

According to the European Atherosclerosis Society consensus statement, triglyceride-rich lipoproteins and their remnants have been implicated in atherosclerotic processes, representing a potential LDL-independent atherogenic pathway contributing to residual cardiovascular risk despite statin therapy [46]. Elevated Remnant C has been observed in the context of hyperinsulinemia, insulin resistance, and inflammatory states. Previous studies have reported associations between elevated triglycerides, Remnant C, chronic low-grade inflammation, and asymptomatic atherosclerosis in the general population, and have suggested greater per-particle atherogenic potential compared with LDL cholesterol [47,48,49,50]. To our knowledge, no prior study has directly examined Remnant C as an independent lipid marker associated with subclinical atherosclerosis in patients with psoriasis.

### 3.3. Immunological and Inflammatory Implications

A key finding of our study is the marked difference in cumulative psoriasis duration between patients with and without atherosclerosis. Patients with CCTA-confirmed stenosis had significantly longer disease duration (median 25 [15, 16, 17, …, 35] vs. 15 [10, 11, 12, …, 27] years). Psoriasis duration remained significantly associated with the presence of atherosclerosis in both univariable (OR 1.059 per year; 95% CI 1.019–1.100; *p* = 0.008) and in multivariable models (Model 1: OR 1.081; 95% CI 1.019–1.147; *p* = 0.010; Model 2: OR 1.069; 95% CI 1.023–1.117; *p* = 0.003). These findings are consistent with the concept of cumulative inflammatory burden as a potential contributor to vascular alterations in psoriasis, suggesting that atherosclerotic burden may be more closely associated with long-term immune stress than with current disease activity. CT-confirmed atherosclerosis in our cohort was associated with a specific immunological pattern not accompanied by differences in classical systemic inflammatory markers. While CRP and erythrocyte sedimentation rate did not differ significantly, IgA levels were significantly associated with atherosclerosis. Patients with stenosis had higher IgA levels, which remained significant predictors in both univariable (OR 1.669; 95% CI 1.002–2.781; *p* = 0.049) and multivariable analyses (OR 1.86; 95% CI 1.04–3.33; *p* = 0.035). In contrast, IgG and IgE levels did not differ between groups, indicating a selective rather than generalized humoral immune activation.

Such findings suggest that IgA may reflect underlying pathophysiological processes related to chronic immune stimulation and subclinical inflammation, potentially through interactions with atherogenic lipoproteins and immune cells within the vascular wall. However, it should be noted that total serum IgA represents a relatively nonspecific marker of immune activation and may be elevated in the context of chronic mucosal immune stimulation, metabolic disturbances, liver dysfunction, or other inflammatory comorbidities. Studies have reported conflicting results regarding the association between elevated inflammatory markers (CRP and erythrocyte sedimentation rate) and PASI scores in patients with psoriasis and asymptomatic atherosclerosis [51,52,53,54]. In contrast to PASI, for which several studies have demonstrated its potential role as an independent predictor of subclinical atherosclerosis, data on the association between CRP and coronary atherosclerosis in patients with psoriasis remain limited and heterogeneous [23]. CRP rarely emerges as a strong and independent predictor of coronary atherosclerosis in patients with psoriasis, particularly in the presence of metabolic disturbances and therapeutic modifiers. The absence of statistical significance in elevated hsCRP values in the group of patients with atherosclerosis may be explained by the fact that hsCRP is an acute-phase reactant and may have limited sensitivity in detecting chronic, low-grade, or compartmentalized immune processes, such as those potentially linking psoriasis and atherosclerosis. Cumulative inflammatory burden over time is difficult to adequately assess through a single time-point measurement of a nonspecific systemic inflammatory marker such as hsCRP.

According to the Rotterdam Study, elevated serum IgA concentrations were independently associated with the presence of severe subclinical atherosclerosis, defined by a high coronary artery calcium score (CAC > 400) [14]. These findings suggest that IgA may reflect specific immunological mechanisms related to atherosclerotic burden, independent of nonspecific systemic inflammation [55]. Furthermore, specific IgA-reactive antibodies, such as anti-malondialdehyde-acetaldehyde (anti-MAA) and anti-β2-glycoprotein I (anti-β2GPI) IgA, have been shown to improve predictive models for atherosclerotic lesions and CAC progression in inflammatory diseases such as rheumatoid arthritis, suggesting a potential involvement of IgA-dependent immune mechanisms in atherosclerotic processes [56,57]. More recent studies have demonstrated that IgA antibodies directed against phosphocholine and polysaccharides of the *Streptococcus pneumoniae* cell wall are associated with increased cardiovascular risk, suggesting a potential role for immune pathways related to gut microbiota and phospholipid metabolism in the pathogenesis of atherosclerosis [58]. One proposed mechanism underlying this association is molecular mimicry, whereby immune responses directed against microbial antigens may cross-react with oxidatively modified phospholipid structures present within atherosclerotic plaques [55]. It should be noted, however, that the above mechanistic insights are primarily based on studies evaluating antigen-specific IgA responses in other inflammatory conditions, whereas the present study assessed total serum IgA.

Based on our targeted literature search, we did not identify studies that directly examined total serum IgA as an independent predictor of subclinical or CT-detected coronary atherosclerosis in patients with psoriasis. Available data in psoriasis populations primarily describe elevated IgA levels and their association with disease activity.

Contemporary concepts of atherogenesis in chronic inflammatory diseases extend beyond the traditional view of isolated risk factors and emphasize a dynamic interaction between metabolic disturbances, atherogenic lipoproteins, and immune mechanisms within a persistent inflammatory milieu. Prolonged cumulative inflammatory burden, characteristic of psoriasis, may over time modulate lipid metabolism, enhance oxidative and glyco-oxidative stress, and promote immune responses that collectively drive the development and progression of atherosclerotic disease. In addition to adaptive immune responses, innate immune mechanisms are increasingly recognized as important contributors to both psoriasis pathogenesis and atherosclerotic cardiovascular disease. Damage-associated molecular patterns (DAMPs), including alarmins such as the S100A8/A9 (calprotectin) complex, have been implicated in chronic inflammatory signaling and vascular inflammation, potentially linking cutaneous immune activation with systemic atherogenesis; however, these pathways were not directly assessed in the present study [59].

Current research by Zappia E. et al. indicates that advanced non-invasive instrumental energy techniques used in dermatological research and clinical practice can modulate inflammatory and metabolic processes and lead to changes in the skin and subcutaneous tissues [60].

## 4. Materials and Methods

A cross-sectional study was conducted in which patients were recruited between May 2024 and December 2025. The study involved 104 patients with chronic severe psoriasis, defined by a Psoriasis Area and Severity Index (PASI) ≥ 10 and/or body surface area (BSA) involvement ≥ 10%. Although the Dermatology Life Quality Index (DLQI) and the involvement of specific anatomical sites were assessed, elevated DLQI scores or specific site involvement were not required for study inclusion.

Eligible participants were consecutive adults (≥ 18 years) with plaque-type psoriasis of at least 3 years’ duration. Patients were allowed to have cardiovascular risk factors; however, exclusion criteria included the presence of clinical cardiovascular symptoms, electrocardiographic abnormalities, or known cardiovascular disease (CVD), defined as a history of coronary artery disease (previous myocardial infarction, stable or unstable angina pectoris, prior percutaneous coronary intervention, or coronary artery bypass grafting), previous cerebrovascular disease (stroke or transient ischemic attack), or peripheral vascular disease. Additional exclusion criteria included the presence of other dermatological diseases, inflammatory autoimmune disorders, or malignancy within the previous 5 years. Patients with active infection, major surgery within the preceding 3 months, pregnancy, or breastfeeding were also excluded.

Patients with contraindications to coronary CT imaging were not included, including significant cardiac arrhythmias (rapid atrial fibrillation or flutter), moderate-to-severe renal impairment (serum creatinine > 133 µmol/L or estimated glomerular filtration rate (eGFR) < 60 mL/min/1.73 m^2^), or a known severe allergic reaction to iodinated contrast media.

Written informed consent was obtained from all participants. The study was conducted in accordance with the Declaration of Helsinki, and the protocol was approved by the Ethics Committee of the University Clinical Center of Serbia (reference number 1038/12, dated 28 March 2024).

### 4.1. Clinical Assessment and Blood Sampling

Clinical data were collected, including medical history, current therapy, anthropometric measurements, electrocardiography, blood pressure values, and heart rate. On the same day, psoriasis severity and extent were assessed using standardized indices, including PASI, BSA, DLQI, and involvement of specific anatomical sites.

Diabetes mellitus, hypertension, and hyperlipidemia were considered present if patients had been previously diagnosed or were receiving appropriate therapy. Diabetes was defined as fasting plasma glucose ≥ 7.0 mmol/L on two separate occasions or HbA1c > 6.5%. Hypertension was defined as a prior diagnosis, use of antihypertensive medication, or systolic blood pressure ≥ 140 mmHg and/or diastolic blood pressure ≥ 90 mmHg (mean of at least two measurements obtained in the seated position on different days). Hyperlipidemia was defined as fasting total cholesterol > 220 mg/dL (5.7 mmol/L), LDL cholesterol ≥ 130 mg/dL, and/or triglycerides > 150 mg/dL (1.7 mmol/L), or the use of lipid-lowering therapy. Obesity was defined as a body mass index (BMI) ≥ 30 kg/m^2^. Smoking status was categorized as current smokers (active smoking or cessation within the past year), former smokers (cessation > 1 year), and non-smokers. A positive family history of CVD was defined as CVD occurring before the age of 55 years in first-degree male relatives or before 65 years in first-degree female relatives.

Venous blood samples were collected from each patient after an overnight fast of at least 8 h. Laboratory analyses were performed in the Center for Medical Biochemistry of the University Clinical Center of Serbia, accredited according to SRPS ISO/IEC 15189:2023 standards [61]. Laboratory parameters included complete blood count with differential, fasting plasma glucose, hemoglobin A1c (HbA1c), lipid profile (total cholesterol, triglycerides, low- and high-density lipoprotein cholesterol), apolipoprotein B (ApoB), and remnant cholesterol (Remnant C), high-sensitivity C-reactive protein (hs-CRP), and immunological parameters, including immunoglobulin A (IgA), immunoglobulin G (IgG), and immunoglobulin E (IgE). Remnant C was calculated as total cholesterol minus the sum of LDL and HDL cholesterol. Remnant C was categorized into tertiles to reduce the influence of extreme values, address potential non-linear associations with outcomes, and enable clinically interpretable risk stratification across concentration ranges. The triglyceride-glucose (TyG) index was calculated as the natural logarithm of the product of fasting triglyceride and fasting glucose concentrations divided by two. The atherogenic coefficient was calculated as the ratio of non-HDL cholesterol to HDL cholesterol. These derived parameters were used to characterize the metabolic–inflammatory and immunological profiles of the participants in relation to the presence of coronary atherosclerosis.

Metabolic syndrome was defined according to the 2009 International Diabetes Federation criteria as the presence of central obesity (waist circumference ≥ 94 cm in men and ≥ 80 cm in women) plus at least two of the following criteria: fasting plasma glucose ≥ 5.6 mmol/L or previously diagnosed diabetes mellitus; blood pressure ≥ 130/85 mmHg or antihypertensive treatment; triglycerides ≥ 1.7 mmol/L or treatment for hypertriglyceridemia; HDL cholesterol < 1.03 mmol/L in men and < 1.29 mmol/L in women.

### 4.2. Coronary Computed Coronary Angiography

In all patients, the assessment of coronary atherosclerosis was based on a combination of coronary computed tomography angiography (CCTA) and coronary artery calcium scoring (CACS). Imaging was performed using a multi-slice multidetector CT scanner with prospective ECG synchronization (slice thickness approximately 2.5–3.0 mm; tube voltage 120 kV). CCTA was conducted following intravenous administration of iodinated contrast media, in accordance with standard clinical protocols, to evaluate the presence of atherosclerotic plaque and luminal narrowing of the epicardial coronary arteries.

CACS was expressed in Agatston units as the sum of calcium scores from the left main coronary artery, left anterior descending artery, circumflex artery, right coronary artery, and posterior descending artery. Coronary atherosclerosis was assessed in accordance with the CAD-RADS 2.0 consensus [62]. Atherosclerosis was defined as the presence of any atherosclerotic plaque on CCTA, regardless of the degree of luminal stenosis, while the absence of plaque was defined as CAD-RADS 0. In cases of extensive coronary calcification limiting accurate luminal assessment, plaque burden was additionally evaluated using CACS as a marker of total atherosclerotic burden. The presence of high CACS values and extensive calcifications involving major epicardial coronary arteries was considered evidence of coronary atherosclerosis. This approach is supported by contemporary evidence emphasizing the important role of CCTA in assessing coronary atherosclerosis in patients with chronic inflammatory diseases, particularly in the presence of extensive coronary calcification that may limit accurate luminal stenosis assessment [63].

### 4.3. Statistical Analysis

Numerical data were presented as mean with a standard deviation, or median with 25th and 75th percentile. Categorical variables were summarized by absolute numbers with percentages. Chi-squared tests, Fisher Exact test, T tests for independent samples and Mann Whiteny U test were used to assess the differences in sociodemographic characteristics, comorbidities, therapy modalities, different risk scores, characteristics of psoriasis, psoriasis therapy, and laboratory data including metabolic parameters, immunologic and inflammatory markers, as well as lipid parameters according to CT-confirmed stenosis in patients with psoriasis. Univariate and multivariate logistic regression models were used to assess predictors of CT-confirmed stenosis. In all analyses, the significance level was set at 0.05. Statistical analysis was performed using IBM SPSS statistical software (SPSS for Windows, release 25.0, SPSS, Chicago, IL, USA). Given the number of univariate comparisons performed, correction for multiple testing was applied using the Benjamini–Hochberg false discovery rate (FDR) method. This approach controls the expected proportion of false discoveries among rejected hypotheses. *p*-values were ordered from smallest to largest, ranked, and adjusted according to the formula *p* × (m/i), where m denotes the total number of tests and i the rank of each *p*-value. The resulting FDR-adjusted *p*-values were interpreted at a significance threshold of 0.05.

Variables included in the multivariable logistic regression models were selected a priori based on clinical relevance, biological plausibility, and prior evidence from the literature. To minimize the risk of overfitting, the number of covariates was limited in accordance with the sample size and number of outcome events, and multicollinearity among candidate variables was assessed prior to model construction. Two multivariable logistic regression models were constructed to evaluate the association between metabolic and clinical factors and CT-confirmed coronary stenosis under different adjustment conditions. Model 1 included demographic and disease-related variables, while Model 2 additionally incorporated treatment-related factors, including statin therapy. This approach was used to explore whether the association between remnant cholesterol and subclinical coronary atherosclerosis remained consistent after adjustment for lipid-lowering treatment, acknowledging the potential for confounding by indication.

## 5. Conclusions

This study demonstrates that patients with psoriasis carry a substantial burden of subclinical atherosclerosis that cannot be fully explained by traditional cardiovascular risk factors. CT-confirmed coronary stenosis was associated with an adverse metabolic, lipid, and immunological profile, highlighting the complex pathophysiology of atherosclerosis in this population. Our findings indicate that metabolic disturbances, particularly insulin resistance and an atherogenic lipid phenotype, play a significant role in the development of coronary atherosclerosis in patients with psoriasis. Remnant C emerged as a strong and independent predictor of coronary atherosclerosis, even after adjustment for age and statin therapy, emphasizing the importance of atherogenic lipoprotein particles beyond the traditional focus on LDL cholesterol. Psoriasis duration represents an important marker of cumulative inflammatory exposure, suggesting that chronic inflammation over time contributes to the progression of coronary disease. Furthermore, the association between IgA levels and the presence of atherosclerosis points toward a potential role of immune activation in psoriasis-related atherogenesis. Collectively, these findings support the concept of psoriasis as a multisystem disease and underscore the need for a broader approach to cardiovascular risk assessment that incorporates metabolic, lipid, and immunological parameters. Our results suggest that Remnant C may represent a significant lipid marker associated with the presence of coronary atherosclerosis in patients with psoriasis, while disease duration highlights the relevance of cumulative inflammatory burden, and the IgA signal opens new avenues for further investigation of immuno–lipid interactions in this population.

We propose that preventive cardiovascular screening should be considered in cardiologically asymptomatic patients with long-standing psoriasis, including assessment of Remnant C, IgA, TyG index, and HbA1c, to identify individuals who may benefit from CCTA for the early detection of coronary atherosclerosis. Such an approach has the potential to improve early risk stratification and, ultimately, reduce cardiovascular morbidity and mortality in patients with psoriasis.

**Limitations:** A limitation of this study is the lack of detailed data regarding duration of statin therapy, and lipid levels prior to statin initiation. Although lipid measurements were obtained at the time of imaging, the absence of information on baseline lipid status and cumulative exposure to lipid-lowering therapy may have influenced the observed associations between lipid parameters and imaging-defined coronary atherosclerosis.

## Figures and Tables

**Table 1:** Sociodemographic characteristics, comorbidities and therapy modalities according to CT-confirmed stenosis in patients with psoriasis.

Variable	CT-Confirmed Stenosis		*p*-Value
	No (*n* = 64)	Yes (*n* = 29)	
**Sociodemographic characteristics, *n* (%)**			
Sex			
Male	42 (65.6)	27 (93.1)	0.005
Female	22 (34.4)	2 (6.9)	
Age, mean ± sd	41.1 ± 11.8	61.4 ± 8.8	<0.001
BMI, mean ± sd	28.7 ± 6.7	31.4 ± 6.2	0.062
Waist circumference, mean ± sd	98.5 ± 16.8	107.8 ± 14.7	0.012
Smoking status			
Non-smoker	18 (28.1)	6 (20.7)	0.031
Ex-smoker	6 (9.4)	9 (31.0)	
Smoker	40 (62.5)	14 (48.3)	
Duration of smoking, median (25th–75th percentile)	15 (9–20)	25 (15–40)	0.005
Family predisposition	18 (28.1)	7 (24.1)	0.688
**Comorbidities, *n* (%)**			
Previous known hypertension	24 (37.5)	20 (69.0)	0.005
Duration of hypertension (months), median (25th–75th percentile)	6 (4–10)	9 (4–15)	0.368
Hyperlipidemia	15 (23.4)	14 (48.3)	0.017
Hypertriglyceridemia	7 (10.9)	16 (55.2)	<0.001
Diabetes mellitus	9 (14.1)	9 (31.0)	0.055
Chronic renal insufficiency	1 (1.6)	1 (3.4)	0.561
Anemia	4 (6.3)	1 (3.4)	0.579
***Therapy***			
Previous antihypertensive therapy	19 (29.7)	21 (72.4)	<0.001
Statins	3 (4.7)	7 (24.1)	0.005
Fibrates	1 (1.6)	5 (17.2)	0.011
Beta blockers	8 (12.5)	13 (44.8)	0.001
ACE Inhibitors and ARBs	16 (25.0)	16 (55.2)	0.005
Thiazide diuretics	3 (4.7)	9 (31.0)	<0.001
Mineralocorticoid receptor antagonists	1 (1.6)	1 (3.4)	0.561
Calcium channel blockers	5 (7.8)	7 (24.1)	0.030

**Table 2:** Characteristics of psoriasis according to CT-confirmed stenosis.

Variable	CT-Confirmed Stenosis		*p*-Value
	No (*n* = 64)	Yes (*n* = 29)	
Age when psoriasis started, median (25th–75th percentile)	20 (16–28)	37 (21–47)	0.001
Psoriasis duration (yrs), median (25th–75th percentile)	15 (10–27)	25 (15–35)	0.008
PASI score	13.1 (10.9–22.7)	12.6 (10.0–16.3)	0.162
BSA	20 (11–45)	20 (12–30)	0.794
DLQI	18 (12–24)	11 (6–18)	0.005
Specific sites affected, *n* (%)	61 (95.3)	28 (96.6)	0.785
EKG, *n* (%)			
Sinus rhythm	63 (100.0)	26 (96.3)	0.125
Atrial fibrillation	0 (0.0)	1 (3.7)	
Metabolic syndrome, *n* (%)	18 (28.1)	19 (65.5)	0.001
Metabolic syndrome score, median (25th–75th percentile)	2 (1–3)	3 (2–4)	<0.001

**Table 3:** Psoriasis therapy according to CT-confirmed stenosis in patients with psoriasis.

Therapy	CT-Confirmed Stenosis		*p*-Value
	No (*n* = 64)	Yes (*n* = 29)	
***Previous therapy, n (%)***			
Topical	63 (98.4)	28 (96.6)	0.561
Systemic	51 (81.1)	22 (75.9)	0.575
Acitretin	26 (41.3)	15 (51.7)	0.349
Metotrexat	35 (55.6)	12 (41.4)	0.206
Biologic	10 (16.1)	3 (10.3)	0.462
***Ongoing therapy, n (%)***			
Systemic	39 (60.9)	25 (86.2)	0.015
Acitretin	15 (23.4)	9 (31.0)	0.438
Metotrexat	14 (21.9)	12 (41.4)	0.052
Biologic	13 (20.3)	4 (13.8)	0.451

**Table 4:** Significant laboratory data according to CT-confirmed stenosis in patients with psoriasis.

Laboratory Data	CT-Confirmed Stenosis		*p*-Value
	No (*n* = 64)	Yes (*n* = 29)	
Total cholesterol *	5.0 ± 1.05	5.35 ± 1.29	0.164
HDL *	1.16 (1.0–1.38)	1.10 (0.90–1.23)	0.100
LDL *	3.10 (2.46–3.49)	2.90 (2.06–3.90)	0.908
CRP *	1.9 (0.8–5.5)	1.5 (1.0–5.0)	0.810
Glc *	4.8 (4.3–5.2)	5.7 (5.2–7.2)	<0.001
HbA1c *	5.3 ± 0.47	6.2 ± 1.1	<0.001
Creatinine *	71 (63.5–82.5)	83 (66–92)	0.021
Tgl *	1.46 (1.07–2.03)	2.09 (1.58–3.28)	0.001
ApoB *	1.05 ± 0.2	1.2 ± 0.3	0.036
ApoA1 *	1.48 ± 0.29	1.44 ± 0.25	0.650
IgA *	2.0 (1.48–2.82)	2.6 (1.06–3.62)	0.045
IgG *	11.4 ± 2.4	11.5 ± 2.7	0.963
IgE *	51 (18–131)	64 (26–168)	0.416
Tyg *	8.59 (8.29–8.97)	9.22 (8.97–9.66)	0.001
Atherogenic Coefficient *	3.09 (2.48–3.95)	3.94 (2.86–4.99)	0.036
Remnant C *	0.65 (0.49–0.89)	0.95 (0.72–1.49)	<0.001

* data presented as mean ± sd or median (25th–75th percentile).

**Table 5:** Univariate logistic regression model with CT-confirmed stenosis in patients with psoriasis as dependent variable.

Variable	FDR-Adjusted *p*-Values	OR	95% CI
Sex, male	0.016	0.141	0.031–0.651
Age	<0.001	1.174	1.101–1.251
Waist circumference	0.019	1.035	1.007–1.065
Duration of smoking	0.013	1.058	1.014–1.104
Hypertension	0.011	3.704	1.453–9.438
Hyperlipidemia	0.021	3.049	1.203–7.728
Hypertriglyceridemia	<0.001	10.022	3.427–29.312
Previous antihypertensive therapy	0.002	6.217	2.345–16.483
Statins	0.015	6.470	1.536–27.243
Beta blockers	0.004	5.687	2.008–16.113
ACE Inhibitors and ARBs	0.010	3.692	1.464–9.312
Thiazide diuretics	0.005	9.150	2.254–37.136
Calcium channel blockers	0.039	3.755	1.078–13.075
Age when psoriasis started	0.002	1.069	1.031–1.109
Psoriasis duration (yrs)	0.008	1.059	1.019–1.100
DLQI	0.011	0.918	0.863–0.977
Glc	0.003	2.095	1.365–3.214
HbA1c	<0.001	6.694	2.474–18.115
Creatinine	0.019	1.035	1.007–1.064
IgA	0.049	1.669	1.002–2.781
Tyg	0.005	3.350	1.550–7.250
Atherogenic Coefficient	0.021	1.431	1.055–1.942
Remnant C	0.004	5.502	1.910–15.848
Metabolic syndrome Score	0.003	1.804	1.280–2.541

*p*-values were adjusted for multiple comparisons using the false discovery rate (FDR) method according to Benjamini–Hochberg.

## Data Availability

The data supporting the findings of this study are available from the corresponding author upon reasonable request. The data are not publicly available due to privacy and ethical restrictions.
